# High-Level Expression of Notch1 Increased the Risk of Metastasis in T1 Stage Clear Cell Renal Cell Carcinoma

**DOI:** 10.1371/journal.pone.0035022

**Published:** 2012-04-10

**Authors:** Qing Ai, Xin Ma, Qingbo Huang, Shangwen Liu, Taoping Shi, Chao Zhang, Mingyang Zhu, Yu Zhang, Baojun Wang, Dong Ni, Hongzhao Li, Tao Zheng, Xu Zhang

**Affiliations:** 1 Medical School, Nankai University, Tianjin, People's Republic of China; 2 State Key Laboratory of Kidney Diseases, Department of Urology, Military Postgraduate Medical College, Chinese People's Liberation Army General Hospital, Beijing, People's Republic of China; The University of Hong Kong, China

## Abstract

**Background:**

Although metastasis of clear cell renal cell carcinoma (ccRCC) is basically observed in late stage tumors, T1 stage metastasis of ccRCC can also be found with no definite molecular cause resulting inappropriate selection of surgery method and poor prognosis. Notch signaling is a conserved, widely expressed signal pathway that mediates various cellular processes in normal development and tumorigenesis. This study aims to explore the potential role and mechanism of Notch signaling in the metastasis of T1 stage ccRCC.

**Methodology/Principal Findings:**

The expression of Notch1 and Jagged1 were analyzed in tumor tissues and matched normal adjacent tissues obtained from 51 ccRCC patients. Compared to non-tumor tissues, Notch1 and Jagged1 expression was significantly elevated both in mRNA and protein levels in tumors. Tissue samples of localized and metastatic tumors were divided into three groups based on their tumor stages and the relative mRNA expression of Notch1 and Jagged1 were analyzed. Compared to localized tumors, Notch1 expression was significantly elevated in metastatic tumors in T1 stage while Jagged1 expression was not statistically different between localized and metastatic tumors of all stages. The average size of metastatic tumors was significantly larger than localized tumors in T1 stage ccRCC and the elevated expression of Notch1 was significantly positive correlated with the tumor diameter. The functional significance of Notch signaling was studied by transfection of 786-O, Caki-1 and HKC cell lines with full-length expression plasmids of Notch1 and Jagged1. Compared to the corresponding controls, all cell lines demonstrated significant promotion in cell proliferation and migration while cell cycle remained unaffected.

**Conclusions/Significance:**

High-level expression of Notch signaling increased the risk of metastasis in T1 stage ccRCC by stimulating the proliferation and migration of tumor cells, which may be helpful for the selection of suitable operation method and prognosis of ccRCC.

## Introduction

Renal cell carcinoma (RCC) is the second leading cause of death among urologic tumors, accounting for 2% of adult malignancies and the most common histologic subtype is clear cell renal cell carcinoma (ccRCC) [1]. Generally, for T1 stage kidney tumor, partial nephrectomy is the recommended surgery for localized RCC and radical nephrectomy is needed for metastatic tumors [2], [3]. However, aside from the imaging evidence, there is no definite molecular targets that can be used to identify the metastatic liability of ccRCC which may help the doctors to determine the suitable operation method in surgery.

In vertebrates there are four Notch genes (Notch1, Notch2, Notch3 and Notch4), which encode receptors for at least five different DSL Notch ligands (Jagged1/Serrate1, Jagged2/Serrate2, Delta1, Delta3 and Delta4). The Notch proteins comprise a conserved, widely expressed family of cell-surface receptors that mediate various cellular processes including differentiation, proliferation, and apoptosis via direct cell-cell interactions [4]. Interestingly, in the development of cancer, Notch may act as either an oncogene or a tumor suppressor gene depending on the tumor type [5].

So far, the mechanism of metastasis in T1 stage ccRCC was poorly understood and there was a contradiction about the expression and function of Notch1 in RCC in two different studies [6], [7], which led us to explore the effect of Notch signaling in the metastasis of early stage ccRCC.

The overall aim of this study was to determine the potential role and mechanism of Notch1 and Jagged1 in the metastasis of T1 stage renal tumors and our data showed that high-level expression of Notch signaling increased the risk of metastasis in T1 stage ccRCC by stimulating the proliferation and migration of tumor cells.

## Materials and Methods

### Ethics Statement

Prior written informed consent was obtained from all patients and the study was approved by the Chinese People's Liberation Army General Hospital's Protection of Human Subjects Committee.

### Patients and tissue samples

51 ccRCC tissue samples including 25 metastatic tumor samples, 26 localized tumor samples and adjacent non-tumorous kidney tissue counterparts used for real-time RT-PCR and Western-blot were collected at Chinese People's Liberation Army General Hospital (Beijing, China). The hard and firm tumor tissues were trimmed free of normal tissue and snap frozen in liquid nitrogen immediately after resection according to the specimen regulation of PLA General Hospital. All RCC cases were clinically and pathologically confirmed to be clear-cell carcinoma and were staged based on the 2011 Union for International Cancer Control (UICC) TNM classification of malignant tumors (Table S1).

### Cell culture and reagents

Human renal proximal tubular epithelial cell line HKC [8], kidney tumor cell line 786-O [9] and Caki-1 [10] were cultured in complete Dulbecco's modified Eagle's medium (Invitrogen, Carlsbad, CA) with 10% fetal bovine serum (Invitrogen), 50 U/ml of penicillin and 50 lg/ml of streptomycin (Invitrogen). All cells were cultured in a sterile incubator maintained at 37°C with 5% CO_2_.

### RNA isolation and real-time RT-PCR

Total RNA was extracted using the PARIS™ Kit (Applied Biosystems, Foster City, CA) according to the manufacturer's protocol. Multiscribe™ Reverse Transcriptase (Applied Biosystems) was used to synthesize the complementary DNA templates. Real-time reverse transcription–polymerase chain reactions were performed in an Applied Biosystems 7500 Detection system using Maxima® SYBR Green/ROX qPCR Master Mix Assays (Fermentas, USA). The expression of mRNA was determined from the threshold cycle (Ct), and the relative expression levels were normalized to the expression of human peptidylprolyl isomerase A (PPIA) mRNA [11] and calculated by the 2^−ΔΔ^Ct method [12]. Primers used in real-time RT-PCR were listed in Table 1.

**Table 1:** Real-time RT-PCR Primers.

Gene	Primer Sequence	Amplicon
Notch1	Forward primer: TGCCAGACCAACATCAAC (18 bp)	192 bp
	Reverse primer: CTCATAGTCCTCGGATTGC (19 bp)	
Jagged1	Forward primer: CCGTTGCTGACTTAGAAT (18 bp)	83 bp
	Reverse primer: AGCCAACCACAGAAACTA (19 bp)	
MMP-9	Forward primer: CCTGGAGACCTGAGAACCAATC (22 bp)	80 bp
	Reverse primer: CCACCCGAGTGTAACCATAGC (21 bp)	
P21	Forward primer: CGCTAATGGCGGGCTG (16 bp)	60 bp
	Reverse primer: CGGTGACAAAGTCGAAGTTCC (21 bp)	
P27	Forward primer: CGCCATATTGGGCCACTAA (19 bp)	89 bp
	Reverse primer: CGCAGAGCCGTGAGCAA (17 bp)	
PPIA	Forward primer: ATGGTCAACCCCACCGTGT (19 bp)	101 bp
	Reverse primer: TCTGCTGTCTTTGGGACCTTGTC (23 bp)	

### Protein extraction and Western-blot analysis

Western blot analysis were carried out as described previously [13]. Protein levels were quantified by Bradford assay. 30 µg protein from each sample was fractionated by 10% sodium dodecyl sulfate polyacrylamide gel electrophoresis and transferred onto polyvinylidene fluoride membranes (PVDF membranes, Millipore). The membrane was blocked in 0.1% Triton X-100 and 5% low fat milk powder in phosphate-buffered saline for 1 hour at 4°C and then probed with anti-glyceraldehyde-3-phosphate dehydrogenase (GAPDH) (1∶500; Santa Cruz Biotechnology, Santa Cruz, CA) rabbit polyclonal primary antibody, anti-Notch1 (1∶500, Abcam, Cambridge, MA) rabbit polyclonal primary antibody, anti-Jagged1 (1∶500, Santa Cruz Biotechnology) rabbit polyclonal primary antibody. After washing 3 times with Tris-buffered saline Tween-20, the membrane was incubated in peroxidase-conjugated goat anti-mouse/rabbit IgG antibody (1∶1000, Santa Cruz Biotechnology). Bands were visualized by an enhanced chemiluminescence detection system using medical X-ray films and quantified by Photoshop (Adobe software). The intensities of band of interest were expressed relative to the GAPDH intensities from the same sample.

### Plasmid transfection

2×10^5^ per well cells were seeded into 60 mm plates 24 hours prior to plasmid transfection. Cells were then transfected with the empty vector (pCMV6-Entry, catalogue No. PS100001, Origene, Rockville, MD), or the human cDNA ORF clone of Notch1 (pCMV6-Notch1, catalogue No. RC211365) and Jagged1 (pCMV6-Jagged1, catalogue No. RC210516) using MegaTran 1.0 (Origene) for 6 hours in OptiMEM I Reduced Serum9 Medium (Invitrogen). Cells were harvested at the indicated time points.

### Cell growth analysis by MTS assay

To assess proliferation, cells were transfected with plasmids as indicated above, 1×10^3^ cells were seeded into 96-well plates in 100 µL of 10% FBS/DMEM and cultured at 37°C in a 5% CO_2_ incubator. After culturing for 24, 48, 72, or 96 hours, 20 µL of the CellTiter 96 Aqueous One Solution (Promega, Madison, WI) was added to each well and then incubated for 1 hour at 37°C in a 5% CO_2_ incubator. Absorbance was measured at 490 nm using a microplate reader.

### Cell migration analysis by transwell assay

Cell migration assay was carried out using Boyden chambers containing Transwell (Corning Costar Corp., Cambridge, MA) membrane filter inserts with pore size of 8 µm. Cells were transfected with plasmids as indicated above. 3×10^4^ cells were then seeded on Boyden chambers (upper chamber) in 150 µl DMEM containing 0.2% BSA. The lower chambers were filled with DMEM containing 10% FBS. After 12 hours of migration at 37°C, cells were stained with crystal violet and counted under a microscope in 5 predetermined fields at ×100 by Photoshop (Adobe software).

### Cell cycle analysis by flow cytometry

48 hours after transfection with plasmids as indicated above, cells were collected, fixed by 70% ethanol for 30 minutes and then washed with ice-cold PBS. For the detection of cell cycle, cell pellets were re-suspended in RNase-containing (1∶100 in dilution) PBS buffer on ice and stained with propidium iodide (PI) (BD Biosciences, San Jose, CA) according to the manufacturer's protocol. Stained cells were analyzed on the FACS-Calibur (BD Biosciences). Data were analyzed using the Cellquest Pro software (BD Biosciences).

### Statistics

When appropriate, two group comparisons were analyzed with a t-test, multiple group comparisons were analyzed with a Dunnett-test, and P values were calculated. P<0.05 was considered significant.

## Results

### Elevated expression of Notch1 and Jagged1 in ccRCC

In order to determine the expression level of Notch1 and Jagged1, 51 ccRCC samples including 25 metastatic tumor samples, 26 localized tumor samples and adjacent non-tumorous kidney tissue counterparts were used for real-time RT-PCR and Western-blot.

**Figure 1:**
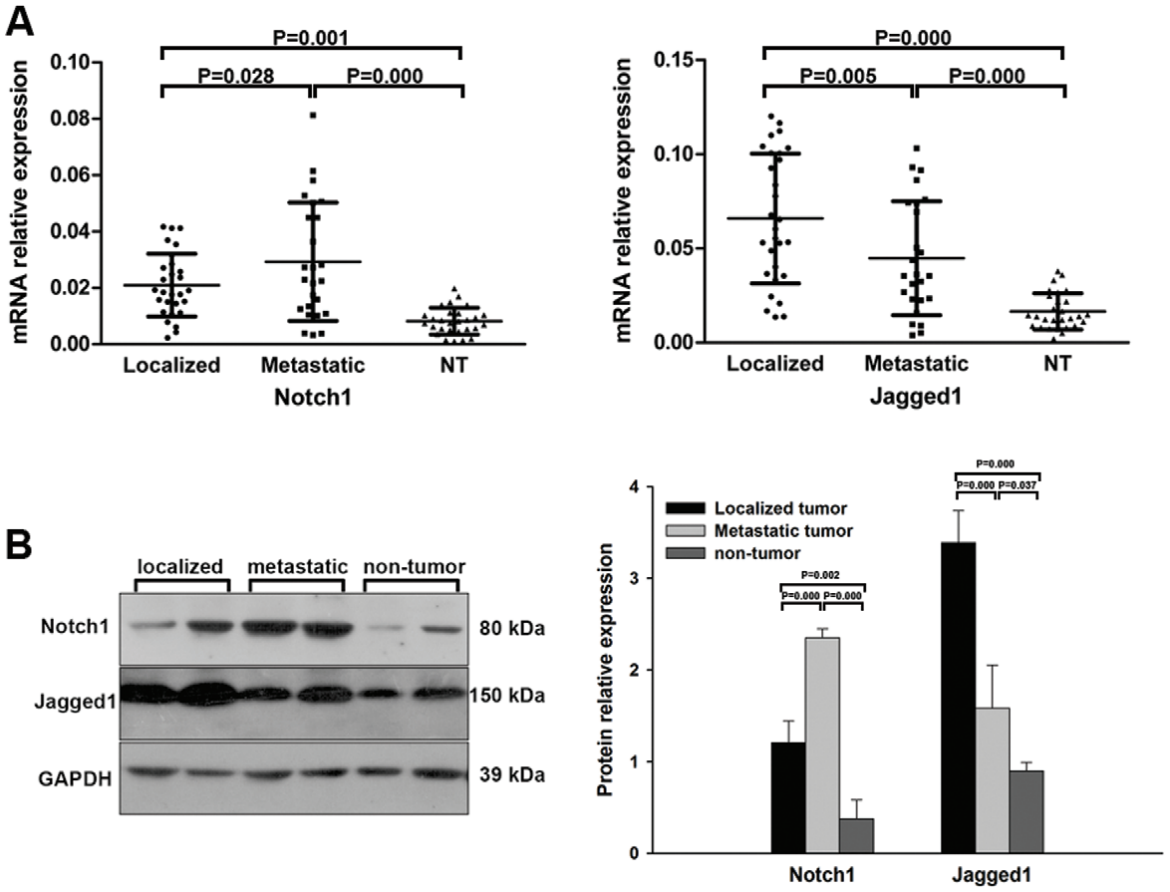
Notch1 and Jagged1 expression in ccRCC tissues. (A) mRNA expression detected by real-time RT-PCR showing elevated Notch1 mRNA expression in localized and metastatic tumors compared to non-tumor(NT) tissues (P = 0.001 and P = 0.000 respectively), and higher expression in metastatic tumors compared to localized tumors (P = 0.028); On the right panel, analysis showing elevated Jagged1 mRNA expression in tumors compared to NT (P = 0.000 and P = 0.000 for localized and metastatic tumors respectively), but lower expression in metastatic tumors compared to localized tumors (P = 0.005). Each dot representing a tissue sample. (B) Protein expression detected by western-blot assay showing elevated expression of Notch1 and Jagged1 protein in tumor tissues compared to non-tumor tissues. The right panel was the densitometric analysis of the bands. The data shown are mean±SD.

After RNA isolation, we performed real-time RT-PCR to measure the mRNA expression level of Notch1 and Jagged1. We found out that Notch1 and Jagged1 expression was significantly elevated in both localized and metastatic tumor tissues compared to non-tumor tissues. Meanwhile Notch1 expression was higher in metastatic tumors but Jagged1 expression was lower compared to localized tumors indicating that the receptor and ligand of Notch signaling may function differently in the metastasis of ccRCC (Figure 1A).

Furthermore, we tested the protein expression in ccRCC samples using western-blot assay. Similar to the mRNA expression level, elevated expression of Notch1 and Jagged1 protein was observed in tumor tissues compared to non-tumor tissues and higher and lower expression of Notch1 and Jagged1 were detected respectively in metastatic tumors compared to localized tumors (Figure 1B).

### High-level expression of Notch1 was associated with metastasis of ccRCC at T1 stage

Based on our clinical experiences and several reports, ccRCC metastasis were sometimes found at T1 or T2 stage [14]. So we next intended to explore the relationship of Notch signaling in the metastasis of early stage ccRCC.

**Figure 2:**
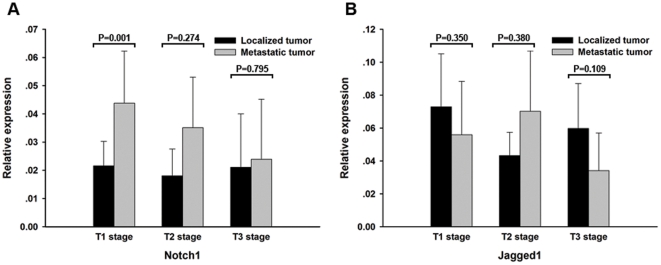
Notch1 and Jagged1 mRNA expression analysis at different stages in ccRCC samples. (A) Analysis showing significantly higher expression of Notch1 in metastatic tumors at T1 stage compared to localized tumors(P = 0.001). (B) No statistically significant difference of Jagged1 expression in all three stages. The data shown are mean±SD.

Tissue samples of localized and metastatic tumors were divided into three groups based on their tumor stages and the relative mRNA expression of Notch1 and Jagged1 were analyzed. As shown in Figure 2A, Notch1 expression was elevated in all stages in metastatic tumors compared to localized tumors but the difference was significant in T1 stage only (P = 0.001). As for Jagged1, the expression level had no statistically significant difference in all three stages (Figure 2B). The results indicated that only the receptor Notch1 was associated with the metastasis of ccRCC at T1 stage.

### Larger tumor size was a risk factor of metastasis of T1 stage ccRCC and correlated with the elevation of Notch1 expression

**Figure 3:**
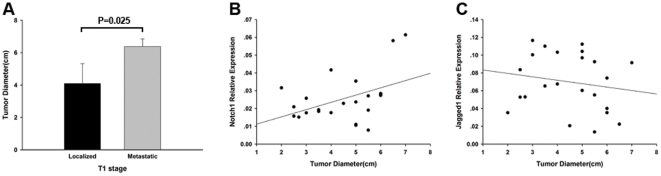
Analysis of tumor diameter and Notch1 and Jagged1 mRNA expression level in T1 stage ccRCC. (A) Analysis showing larger average diameter in metastatic tumors (6.375±0.479 cm n = 4) compared to localized tumors (4.089±1.237 cm, n = 19, P = 0.025). (B) Positive correlation of Notch1 expression and tumor diameter in T1 stage(n = 23, R = 0.435, P = 0.038). (C) No correlation of Jagged1 expression and tumor size in T1 stage(n = 23, R = −0.172, P = 0.432). The data shown are mean±SD.

Renal tumor size has been reported by groups from multiple institutions to be significantly associated with the risk of synchronous and asynchronous metastasis [15], [16]. So next we analyzed the tumor diameter of localized and metastatic tumors in T1 stage to find out whether larger tumor size could be a risk factor for metastasis of early stage ccRCC. As shown in Figure 3A, the average size of metastatic tumors was 6.375±0.479 cm (n = 4) in diameter which was larger than localized tumors (4.089±1.237 cm, n = 19, P = 0.025). Furthermore, in order to determine whether Notch signaling was relevant to the tumor size, we analyzed the correlation of Notch1 and Jagged1 expression and tumor diameter in T1 stage ccRCC. As shown in Figure 3B and 3C, Notch1 expression was positive correlated with tumor diameter (n = 23, R = 0.435, P = 0.038) while Jagged1 expression was not significantly correlated with tumor size (n = 23, R = −0.172, P = 0.432).

### Forced expression of Notch1 and Jagged1 stimulated the proliferation of tumor cell

Since the tumor size was mainly determined by the growth ability of tumor cells and Notch1 expression was positive correlated with tumor size in T1 stage, we next intended to verify whether larger tumor size which was observed in metastatic ccRCC was a result of high-level expression of Notch signaling.

After transfecting Caki-1, 786-O and HKC cell lines with pCMV6-Notch1, pCMV6-Jagged1 and pCMV6-Entry as indicated above, we examined the cell proliferation by MTS assay.

**Figure 4:**
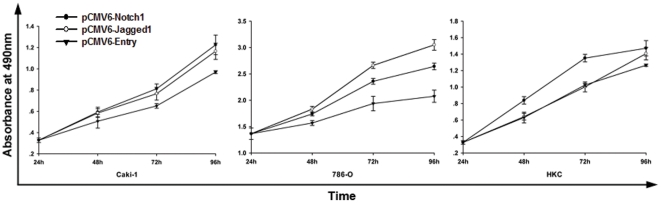
Notch1 promoting the proliferation in tumor and normal kidney cell lines. Proliferation assay by MTS showing increased proliferation in Caki-1 and 786-O cell line after Notch1 and Jagged1 expression. MTS assay in HKC cell line showing increased proliferation rate in Notch1 expressing cells only. The data shown are means±SD from two independent experiments, each carried out in triplicate.

As shown in Figure 4, forced expression of Notch1 and Jagged1 promoted the growth of Caki-1 and 786-O cell line. For HKC cell line, over expression of Notch1 also stimulated the growth ability but there was no significant change on cell proliferation when transfected with Jagged1 plasmid. The results showed that aside from maintaining the growth of normal kidney cells, Notch1 could also stimulate the growth of tumor cells which may facilitate the progression of early stage ccRCC.

### Forced expression of Notch1 and Jagged1 promoted the migration of tumor cell

Elevated cell migration is an important mechanism that is thought to increase metastatic potential of cancer cells. This maybe independent of cell proliferation rates. Therefore, we studied the effect of Notch1 and Jagged1 on cell migration of 786-O and Caki-1 cell lines using the Boyden chamber transwell assay.

**Figure 5:**
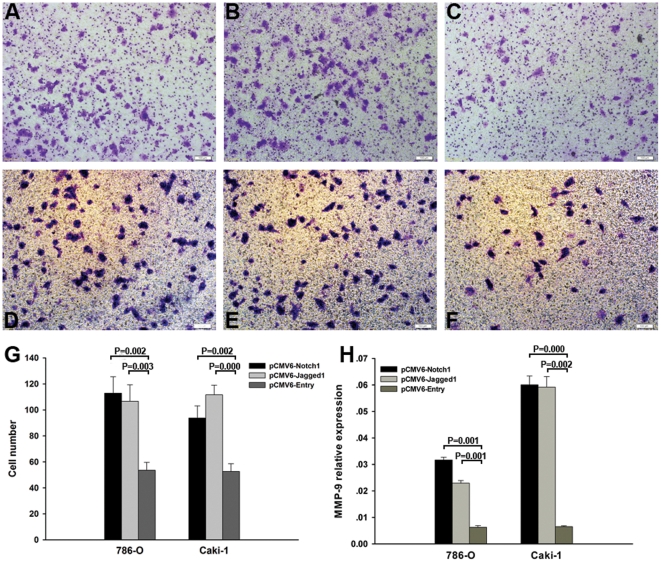
Notch1 and Jagged1 promoting the migration in tumor cell lines. (A)–(C) are representative view of 786-O cell line transfected with Notch1, Jagged1 and control plasmid respectively. (D)–(F) are Caki-1 cell line transfected with Notch1, Jagged1 and control plasmid respectively. (G) Notch1 and Jagged1 can promote the migration of 786-O and Caki-1 tumor cell lines compared to the controls. (H) MMP-9 mRNA expression was significantly elevated in Caki-1 and 786-O cell lines after transfected with Notch1 and Jagged1 plasmid. The data shown are mean±SD.

For 786-O cell line, after transfected with pCMV6-Notch1, pCMV6-Jagged1 and pCMV6-Entry, the number of cells that migrated through the Transwell membrane was 112.8±12.7, 106.6±12.7, and 53.6±6.0 (P = 0.002 and P = 0.003, respectively, Figure 5A–C,G). For Caki-1 cell line, the number of cells that migrated through the Transwell membrane was 93.8±9.3 for Notch1 expression cells, 111.6±7.4 for Jagged1 expression cells, and 52.6±5.9 for control cells (P = 0.002 and P = 0.000, respectively, Figure 5D–F). To further verify the pro-metastatic function of Notch signaling, we next examined the expression change of MMP-9 which is a known metastasis gene [17] after forced expression of Notch1 and Jagged1. As shown in Figure 5H, the mRNA expression of MMP-9 was significantly elevated in both 786-O and Caki-1 cell lines after over-expression of Notch1 and Jagged1. Taken together, the results indicated that high-level expression of Notch signaling may contribute to the metastasis of ccRCC by promoting the migration ability of tumor cells.

### Notch1 and Jagged1 did not affect the cell cycle of tumor cell

**Figure 6:**
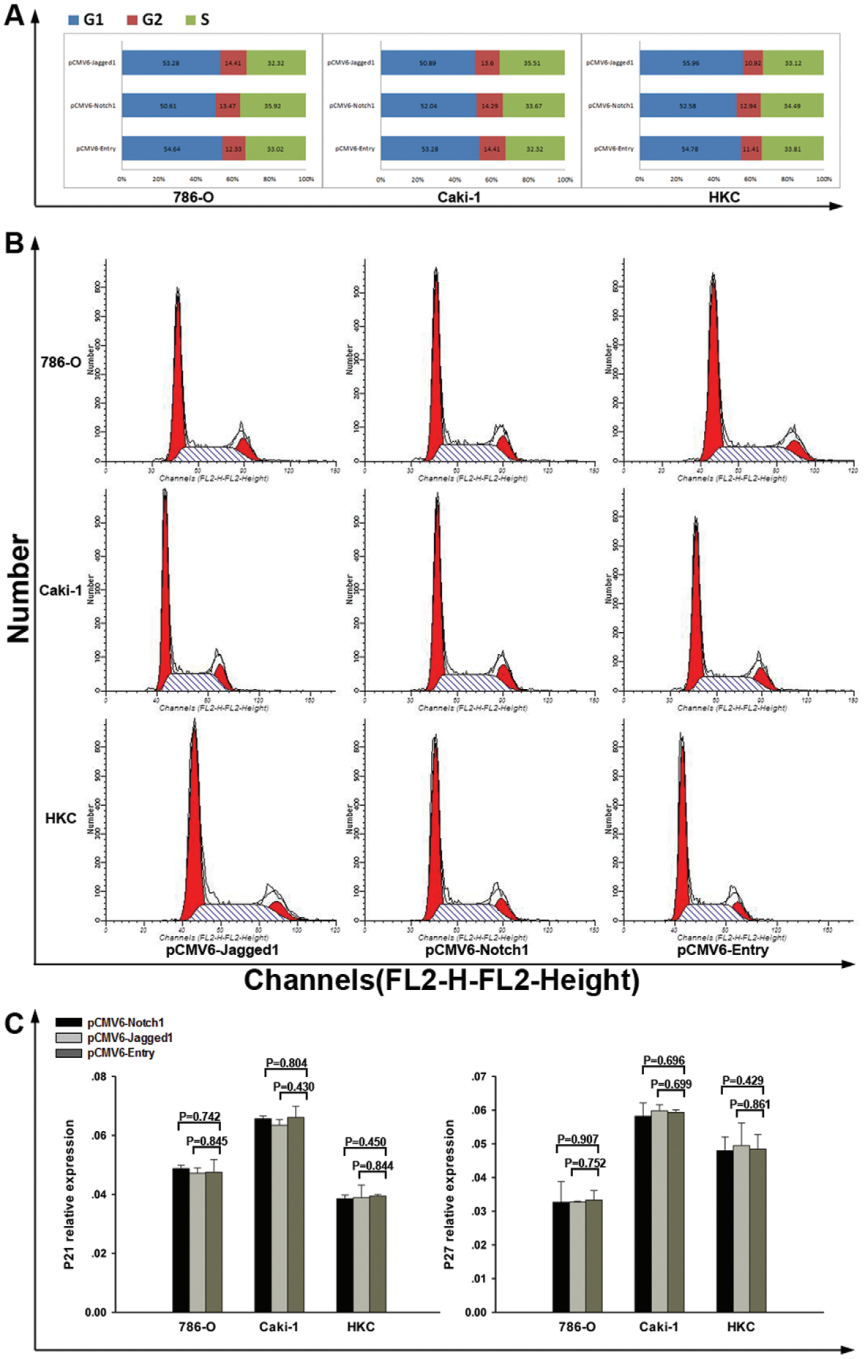
Analysis of cell cycle with over-expression of Notch1 and Jagged1. (A)–(B) In 786-O, Caki-1 and HKC cell line, cell cycle analysis demonstrating no difference between cells over-expressing Notch1 and Jagged1 compared to controls. (C) P21 and P27 remained unchanged in all cell lines after transfected with Notch1 and Jagged1 plasmids.

To determine whether high-level expression of Notch signaling could induce cell cycle alteration, we ectopic expressed pCMV6-Notch1, pCMV6-Jagged1 and pCMV6-Entry in 786-O, Caki-1 and HKC cell lines and detected the cell cycle by flow cytometry. As shown in Figure 6A–B, over expression of Notch1 and Jagged1 did not lead to significant change of cell cycle in all cell lines. Furthermore, we examined the expression of P21 and P27 to verify the negative affection on cell cycle. As shown in Figure 6C, both P21 and P27 mRNA expression remained unchanged after over-expression of Notch1 and Jagged1 indicating that the pro-metastatic effect of Notch signaling may not function through the regulation of cell cycle in ccRCC.

## Discussion

Metastasis, the spread of tumor cells from the site of origin to distant organs, has long been recognized as the major threat to the survival of cancer patients [18], [19]. Localized T1 stage ccRCC is recommended for partial nephrectomy [3], however, distant metastasis after the surgery was detected occasionally resulting a second operation to remove the kidney and poor prognosis of the patient. So far, the mechanism of metastasis was poorly understood [20], thus it is imperative to find effective molecular targets for the diagnosis and prevention of early stage kidney cancer metastasis.

Although Notch1 has been reported to be pro-metastatic in multiple cancers [21]–[25], the exact role of Notch signaling in kidney cancer is not clear yet, so we intended to explore the function of Notch signaling in the metastasis of ccRCC. First, we studied the basic expression level of Notch1 and Jagged1 in 25 metastatic tumors, 26 localized tumors and adjacent non-tumorous kidney tissue counterparts. Compared to non-tumor tissues, Notch1 and Jagged1 expression were significantly elevated both in mRNA and protein levels in localized and metastatic tumors which was consistent with some other reports [6], [26]. However, some investigators had also given opposite results that the expression of Notch receptors was down-regulated and Notch signaling might function as a tumor suppressor in the progress of RCC [7]. Since Notch signaling is such a complicated and comprehensive pathway, the underlying detailed mechanism of this contradictory requires further study including an extensive and complete study of all the receptors, ligands and even downstream target genes.

Next we analyzed the mRNA expression of Notch1 and Jagged1 on different tumor stages of ccRCC. Compared to localized tumors, Notch1 expression was significantly elevated in metastatic tumors in T1 stage only while Jagged1 expression was not statistically different among all stages indicating that only the receptor Notch1 was associated with the metastasis of ccRCC at T1 stage.

Since tumor size had been reported to be a risk factor in the metastasis of T1 stage RCC [27], [28], we analyzed the correlation of tumor size and relative mRNA expression of Notch1 and Jagged1 in 23 T1 stage ccRCC samples. Notch1 expression was positive correlated with tumor diameter while Jagged1 expression had no significantly correlation with tumor size.

Taken together, we postulated that Notch1 may stimulate the growth of tumor cells resulting larger tumor size and finally metastasis of early stage ccRCC.

In order to verify our postulation, we transfected tumor cell lines with Notch1 plasmid and examined the alteration of tumor cell proliferation. As expected, the growth ability was significantly enhanced after transfection indicating that elevated proliferation by Notch1 was indeed a pivotal element of metastasis in early stage tumor. Aside from the growth promoting function, we further discovered Notch1 could also stimulate the migration of tumor cells which will definitely facilitate the metastasis of kidney tumors.

Similar to Notch1, forced expression of Jagged1 also stimulated the proliferation and migration of tumor cells. Because of the function of Jagged1 which could enable the metastasis of ccRCC, it is hard to understand the difference between Jagged1 and Notch1 such as relative lower expression of Jagged1 in metastatic tumors compared to localized tumors. The inconsistence of expression level and function of Jagged1 may be attributed to the complexity of regulatory mechanism in vivo [29]. Further study is needed in the future to explicit the mechanism including interaction between the receptor and ligand and potential regulator of Notch signaling.

In conclusion, high-level expression of Notch signaling is associated with the metastasis of T1 stage ccRCC by stimulating the proliferation and migration of tumor cells, providing a new angle for the treatment and prevention of kidney cancer in clinic.

## Supporting Information

Table S1 **Tumor samples parameters.**
(DOC)
