# Supplementary material for: High-Level Expression of Notch1 Increased the Risk of Metastasis in T1 Stage Clear Cell Renal Cell Carcinoma
Source: PLoS One. 2012 Apr 10;7(4):e35022. doi: 10.1371/journal.pone.0035022 (PMC3323638; doi:10.1371/journal.pone.0035022)
Supplement: Table S1 — Tumor samples parameters. (DOC) [file pone.0035022.s001.doc]

# Table S1

| Number | Gender | Age(year) | Tumor Diameter(cm) | Tumor Stage | Metastasis |
| --- | --- | --- | --- | --- | --- |
| 1 | male | 73 | 5.2 | T1 | no |
| 2 | female | 67 | 2.8 | T1 | no |
| 3 | male | 55 | 5.6 | T1 | no |
| 4 | male | 48 | 5.7 | T1 | no |
| 5 | female | 57 | 4.7 | T1 | no |
| 6 | male | 51 | 3.3 | T1 | no |
| 7 | male | 48 | 4.6 | T1 | no |
| 8 | male | 63 | 4.4 | T1 | no |
| 9 | male | 54 | 9.9 | T2 | no |
| 10 | male | 45 | 6 | T1 | no |
| 11 | female | 45 | 5.6 | T1 | no |
| 12 | male | 61 | 4.2 | T1 | no |
| 13 | male | 48 | 4 | T1 | no |
| 14 | male | 41 | 6 | T1 | no |
| 15 | female | 41 | 11 | T3 | no |
| 16 | female | 40 | 7 | T2 | no |
| 17 | male | 46 | 2.6 | T1 | no |
| 18 | male | 41 | 6 | T3 | no |
| 19 | male | 64 | 11.5 | T3 | no |
| 20 | male | 33 | 3.3 | T1 | no |
| 21 | male | 41 | 3.7 | T1 | no |
| 22 | male | 63 | 5.5 | T1 | no |
| 23 | male | 43 | 5.5 | T3 | no |
| 24 | male | 54 | 13.1 | T3 | no |
| 25 | male | 49 | 5.2 | T1 | no |
| 26 | male | 46 | 2.88 | T1 | no |
| 27 | male | 61 | 11.6 | T3 | yes |
| 28 | male | 56 | 7.3 | T1 | yes |
| 29 | male | 67 | 6.5 | T3 | yes |
| 30 | female | 52 | 5.2 | T1 | yes |
| 31 | male | 49 | 10 | T3 | yes |
| 32 | male | 65 | 8.7 | T2 | yes |
| 33 | male | 25 | 17.3 | T3 | yes |
| 34 | male | 47 | 9.5 | T2 | yes |
| 35 | male | 47 | 6.6 | T1 | yes |
| 36 | male | 45 | 7.6 | T1 | yes |
| 37 | male | 48 | 10.7 | T3 | yes |
| 38 | male | 36 | 14 | T3 | yes |
| 39 | male | 43 | 15.4 | T2 | yes |
| 40 | male | 22 | 10 | T3 | yes |
| 41 | male | 52 | 8.4 | T2 | yes |
| 42 | female | 36 | 10 | T3 | yes |
| 43 | male | 49 | 12.3 | T3 | yes |
| 44 | female | 22 | 10.5 | T3 | yes |
| 45 | female | 37 | 15 | T3 | yes |
| 46 | male | 49 | 13 | T2 | yes |
| 47 | male | 57 | 13.8 | T3 | yes |
| 48 | male | 56 | 11.8 | T3 | yes |
| 49 | male | 58 | 9.5 | T3 | yes |
| 50 | male | 51 | 11 | T3 | yes |
| 51 | male | 49 | 6.9 | T3 | yes |
